# Comparing mail-in self-collected specimens sent via United States Postal Service versus clinic-collected specimens for the detection of *Chlamydia trachomatis* and *Neisseria gonorrhoeae* in extra-genital sites

**DOI:** 10.1371/journal.pone.0189515

**Published:** 2017-12-14

**Authors:** Katheryn R. Salow, Adam C. Cohen, Claire C. Bristow, Mark R. McGrath, Jeffrey D. Klausner

**Affiliations:** 1 Public Health Division, AIDS Healthcare Foundation, Los Angeles, California, United States of America; 2 Department of Medicine, University of California San Diego, La Jolla, California, United States of America; 3 David Geffen School of Medicine, University of California Los Angeles, Los Angeles, California, United States of America; 4 Fielding School of Public Health, University of California Los Angeles, Los Angeles, California, United States of America; Fred Hutchinson Cancer Research Center, UNITED STATES

## Abstract

**Objectives:**

To evaluate the concordance between clinic-collected extra-genital specimens and self-collected mailed-in extra-genital specimens among participants seeking sexually transmitted infection testing at a free clinic in Hollywood, CA.

**Methods:**

A convenience sample of 210 men who have sex with men were enrolled between February 29, 2016 and December 21, 2016 and received mail-in testing kits for *Chlamydia trachomatis* (CT) and *Neisseria gonorrhoeae* (NG). All testing was performed using the GeneXpert® CT/NG (Cepheid, Sunnyvale, CA).

**Results:**

From the 210 mail-in kits distributed, 149 mail-in kits (71.0%) were returned to the laboratory, resulting in 145 pairs (clinic-collected and mail-in) of rectal test results and 148 pairs of pharyngeal test results for both CT and NG detection. The concordance was 95.0% for all CT rectal tests, 99.3% for all CT pharyngeal tests, 95.7% for all NG rectal tests, and 97.2% for all NG pharyngeal tests.

**Conclusion:**

Roughly two-thirds of mail-in test kits were returned and concordance was generally high, however more than one-third of positive results were missed in mail-in samples. The prevalence of potential false-negative results among mail-in samples warrants caution when implementing mail-in STI testing strategies.

## Introduction

*Chlamydia trachomatis* (CT) and *Neisseria gonorrhoeae* (NG) infections are common and frequently asymptomatic sexually transmitted infections (STIs) and therefore often go undiagnosed and untreated [1–2]. The United States experienced nearly 1.6 million CT cases and nearly 500,000 NG cases in 2016. In Los Angeles County—the largest county in the United States—rates of STIs reached record highs at 581.3 per 100,000 population for CT and 220.0 per 100,000 population for NG [3]. Los Angeles has the highest number of CT and NG cases of any county in the United States [3].

The CDC recommends anatomic site-specific diagnostic tests for CT/NG. Studies evaluating self-collected CT/NG site-specific swab tests—which are easy for patients to use in non-clinical settings—show promising results in terms of their ability to detect previously undiagnosed and untreated CT/NG infections [4–5]. STI testing clinics that distribute CT/NG site-specific swabs to be used outside the clinic setting may allow patients to self-collect rectal and pharyngeal swab specimens in the privacy of their own homes and mail specimens to the laboratory. In-home self-collected specimens may increase the utilization of anatomic site-specific testing for CT and NG infections. As public health professionals promote patients’ self-collection of specimens as part of STI testing, in-home self-collected specimens that can be mailed to the laboratory by the patient represents a critical step forward in ensuring high-risk patients have access to regular STI testing [6–9].

The primary aim of this study was to evaluate the concordance between clinic-collected extra-genital specimens and extra-genital specimens sent via United States Postal Service (USPS) from participants seeking STI testing at a free clinic in Hollywood, CA.

## Methods

A convenience sample of men who have sex with men (MSM) who sought free STI testing at a Hollywood, CA clinic were enrolled between February 29, 2016 and December 21, 2016 and received mail-in testing kits for CT and NG. The clientele that visit this clinic were predominantly MSM that engage in high-risk sexual behaviors. Recruitment occurred five days per week for the duration of the study.

Clinic-based rectal swab specimens were self-collected by the participant in accordance with the clinic’s standard of care, while pharyngeal swab specimens were collected by trained research associates. For mail-in specimen collection, participants were asked to self-collect rectal and pharyngeal swab specimens at home after leaving the clinic and then mail those samples via USPS to the laboratory at their earliest convenience (within 48 hours). Participants who were projected to receive presumptive treatment while at the clinic (i.e., treatment on the same day as the test, but prior to a positive test result) were asked to self-collect the mail-in swabs before leaving the clinic to ensure treatment would not affect the swab specimens. All testing was performed using the GeneXpert® CT/NG (Cepheid, Sunnyvale, CA) by trained laboratory staff. Multiple studies have successfully evaluated the sensitivity and specificity of the GeneXpert® CT/NG (Cepheid, Sunnyvale, CA) compared to other nucleic acid amplification test (NAAT) platforms for screening extra-genital infections [10–14].

As part of the self-collection package, participants were provided written and visual instructions, two swabs in sealed containers (one swab per anatomic site, labeled “T” for throat/pharyngeal, and “R” for rectal), one biohazard bag, and one envelope stamped and addressed to the laboratory. During self-collection, participants were instructed to open one container at a time, remove the swab, swab their designated anatomic site, place the used swab back in the container, close the container, and place the container in the biohazard bag. After both swabs were utilized, participants were instructed to seal the biohazard bag, place the biohazard bag in the envelope, seal the envelope, and place the envelope in a USPS mailbox. Participants received a $25.00 electronic gift card once their mail-in test kit was received. For mail-in specimens, participants were each provided dry FLOQSwabs™ (COPAN Diagnostics, Inc., Murrieta, CA) to avoid transporting fluid via USPS. Once our lab received the mailed specimens, the FLOQSwabs™ were transferred to a vial from the Xpert® Vaginal/Endocervical Specimen Collection Kit. This vial included liquid transport reagent which hydrated the dry swabs prior to running these samples on the GeneXpert® CT/NG. Xpert® Vaginal/Endocervical Specimen Collection Kit with liquid transport reagent were used for clinic-collected specimens. Studies by Gaydos et al. and Coorevits et al. have validated the concordance of dry swabs to wet swabs in CT/NG screening platforms [15–16].

Pairs of clinic-collected specimens and mail-in specimens were analyzed to determine the concordance (percent agreement), and 95% confidence intervals (CI) were calculated using exact binomial methodology.

The GeneXpert® CT/NG assay includes a control mechanism called the sample adequacy control (SAC) that targets a single copy human gene, hydroxymethylbilane synthase, to control for false negative results where no human cells are present by confirming adequate patient sample has been collected. The SAC cycle threshold quantifies the number of cycles required to detect the presence of this human gene target. A lower SAC cycle threshold value indicates an earlier cycle detection threshold and more human cellular target in the specimen. If the target is not present, the SAC will fail and the results will be invalid. We compared the SAC cycle threshold values between mail-in and clinic-collected specimen tests using the Wilcoxon signed rank sum test. All analyses were conducted using SAS version 9.4 (Cary, NC).

Ethical approval for this study was granted by the Institutional Review Board (IRB) at the University of California Los Angeles (IRB #15–000740). The IRB waived the requirement for signed informed consent for this research under 45 CFR46.117(c)(2). Verbal informed consent was obtained from all participants. Research associates documented that consent was obtained by initialing next to each participant’s assigned study number on the recruitment document. Each participant received a copy of the information sheet describing the study.

## Results

From the 210 mail-in kits distributed, 149 kits (71.0%) were returned to the laboratory via USPS—however, one returned kit did not contain a pharyngeal swab, and four clinic-collected rectal swabs were lost during transportation from the clinic to the laboratory. There were 145 pairs (clinic-collected and mail-in) of rectal test results and 148 pairs of pharyngeal test results for both CT and NG detection. Table 1 provides a summary of the test results of those pairs by anatomic site separated by collection location (clinic-collected versus mail-in). Eight (2.7%) tests gave invalid results on the GeneXpert® CT/NG. All of the invalid results were due to a SAC failure, indicating the samples contained no human DNA—likely due to insufficient mixing of the sample or an inadequately taken sample [17]. One of these invalid results also reflected a specimen processing control failure in addition to the sample adequacy control failure, indicating improper sample processing within the GeneXpert® CT/NG.

**Table 1:** Comparison of clinic-based specimen collection and mail-in specimen collection on Xpert for detection of CT and NG in rectal and pharyngeal samples.

**A. Rectal*Neisseria gonorrhoeae*.**					
		*Clinic-based result*			Total
		Positive	Negative	Invalid	
*Mail-in result*	Positive	12	0	0	12
	Negative	6	123	1	130
	Invalid	0	3	0	3
Total		18	126	1	145
**B. Pharyngeal*Neisseria gonorrhoeae*.**					
		*Clinic-based result*			Total
		Positive	Negative	Invalid	
*Mail-in result*	Positive	11	0	1	12
	Negative	3	131	0	134
	Invalid	2	0	0	2
Total		16	131	1	148
**C. Rectal*Chlamydia trachomatis*.**					
		*Clinic-based result*			Total
		Positive	Negative	Invalid	
*Mail-in result*	Positive	11	0	1	12
	Negative	7	123	0	130
	Invalid	0	3	0	3
Total		18	126	1	145
**D. Pharyngeal*Chlamydia trachomatis*.**					
		*Clinic-based result*			Total
		Positive	Negative	Invalid	
*Mail-in result*	Positive	1	1	0	2
	Negative	0	143	1	144
	Invalid	1	1	0	2
Total		2	145	1	148

Table 2 provides a summary of the concordance between clinic-collected specimens and mail-in specimens by anatomic site. For CT, the concordance was 95.0% (95% CI: 90.0, 98.0) for all rectal tests, with 61.1% (95% CI: 35.8, 82.7) agreement among positive samples and 100.0% (95% CI: 97.1, 100.0) agreement among negative samples. The concordance was 99.3% (95% CI: 91.0, 98.4) for all pharyngeal tests, with 100.0% (95% CI: 2.5, 100.0) agreement among positive samples and 99.3% (95% CI: 96.2, 100.0) agreement among negative samples.

**Table 2:** Concordance between in-home self-collected and clinic-collected CT/NG specimens using the GeneXpert® CT/NG.

		Positive	Negative	Concordance
		Agreement	Agreement	
		(CI)	(CI)	(CI)
***Chlamydia trachomatis***	Rectal	61.1%	100%	95.0
		(35.8, 82.7)	(97.1, 100.0)	(90.0, 98.0)
	Pharyngeal	100.0%	99.3%	99.3%
		(2.5, 100.0)*	(96.2, 100.0)	(96.2, 100.0)
***Neisseria gonorrhoeae***	Rectal	66.7%	100.0%	95.7%
		(41.0, 86.7)	(97.1, 100.0)	(91.0, 98.4)
	Pharyngeal	78.6%	100%	97.2%
		(49.2, 95.3)	(97.2, 100.0)	(93.1, 99.2)

*There was only one positive CT in this category.

95% confidence intervals were calculated using the exact binomial method.

For NG, the concordance was 95.7% (95% CI: 91.0, 98.4) for all rectal tests, with 66.7% (95% CI: 41.0, 86.7) agreement among positive samples and 100.0% (95% CI: 97.1, 100.0) agreement among negative samples. The concordance was 97.2% (95% CI: 93.1, 99.2) for all pharyngeal tests, with 78.6% (95% CI: 49.2, 95.3) agreement among positive samples and 100.0% (95% CI: 97.2, 100.0) agreement among negative samples.

We were able to retrieve SAC cycle threshold data for 131 out of 145 pairs of rectal swabs. The median cycle threshold for mail-in rectal specimen was 33.2 (Interquartile range (IQR): 28.8, 36.4) compared to the median cycle threshold for clinic-collected rectal specimen was 24.65 (22.7, 29.9) (p<0.0001). We retrieved the SAC cycle threshold data for 135 out of 148 pharyngeal swabs. The median SAC cycle threshold was 29.1 (IQR: 27.2, 30.9) for pharyngeal mail-in samples, while the median cycle threshold for clinic-collected pharyngeal specimen was 24.6 (IQR: 23.2, 25.7) (p<0.0001). All relevant data files are available from Zenodo database (DOI: 10.5281/zenodo.1069568).

## Discussion

We evaluated the concordance between clinic-collected specimens and mail-in specimens for detection of CT and NG infections and found that concordance was generally high. However, more than one-third of positive results were missed in self-collected mail-in samples. Similar high rates of discordance among positive results were seen in a study published by Owens et al. evaluating accuracy of home-based STI testing [18].

Receipt of mailed samples showed optimistic results. Roughly two-thirds of mail-in test kits were returned, a return rate higher than those reported in similar studies that evaluated mail-in STI screening strategies [8,19]. This return rate was particularly encouraging considering no follow-up communication between the research associates and the study participants occurred.

There were several limitations in this study. First, this study utilized a convenience sample of participants in Hollywood, CA and the results may not be generalizable to other populations. Second, this study could have benefitted from an FDA-approved reference test, however, currently no NAATs are FDA-approved for use with extra-genital specimen types. Third, due to the small sample size of participants who received positive results, our confidence intervals for concordance of positive results are wide. Finally, there is a degree of uncertainty when comparing samples collected with the dry FLOQSwabs™ to those collected with the Xpert® Vaginal/Endocervical Specimen Collection Kit with liquid transport reagent. While previous research has validated their concordance using NAAT based CT/NG screening, none have evaluated their concordance using the Cepheid GeneXpert® CT/NG in extra genital sites [15–16]. It is possible the different swab types could explain some of the discordance between clinic-collected and mailed-in samples.

A study conducted by Bristow et al. found that lower SAC cycle threshold values were associated with CT and NG infections in urine samples, suggesting that increased amounts of human cellular materials were correlated with infection status [20]. The higher median cycle threshold value we found in mail-in samples indicates that specimen sent via USPS may have contained significantly less human DNA than clinic-collected specimen. This could have influenced the results and might explain the high frequency of discordant rectal samples.

The prevalence of the potential for false-negative results among self-collected mail-in samples warrants caution when implementing mail-in STI testing strategies. Given that both clinic-collected and mail-in rectal samples were self-collected by the patients, the seven discordant CT and six discordant NG results for mailed-in rectal samples are likely indicative of an inadequacy of self-collection when patients are collecting the swabs outside of the clinic setting. Additionally, there is a possibility that the process of sending dry swab specimens via USPS yields results less consistent than those maintained in the clinic.

While the present study indicates that mail-in STI testing may not be a viable alternative to clinic-based STI testing, focus groups suggest people prefer having the option to test in the comfort of their own homes [8,21–24]. Graseck et al. evaluated the completion rates of STI screening at home versus in a clinic [8]. The researchers found that, when given a choice, more women elected for home-based testing, and those who chose home-based testing were more likely to complete a CT or NG test compared to all clinic-based testers [8]. Their research suggests that mail-in collection strategies may reach a greater number of individuals for screening that otherwise would not access testing services.

Future research should analyze the cost-effectiveness of mail-in samples versus clinic-collected samples due to the low cost of mailing specimens. In addition, providing easy-to-use in-home self-collected test kits may address access, confidentiality, and stigma-related barriers to STI clinic-based services. As STI rates continue to rise in Los Angeles County and throughout the United States, it is imperative for public health professionals to find novel approaches to ensure high-risk patients partake in regular STI testing.
